# Tubulin acetylation enhances lung cancer resistance to paclitaxel-induced cell death through Mcl-1 stabilization

**DOI:** 10.1038/s41420-021-00453-9

**Published:** 2021-04-06

**Authors:** Onsurang Wattanathamsan, Rawikorn Thararattanobon, Ratchanee Rodsiri, Pithi Chanvorachote, Chanida Vinayanuwattikun, Varisa Pongrakhananon

**Affiliations:** 1 Inter-department Program of Pharmacology, Graduate School, Chulalongkorn University, Bangkok, 10330 Thailand; 2 Preclinical Toxicity and Efficacy Assessment of Medicines and Chemicals Research Cluster, Chulalongkorn University, Bangkok, 10330 Thailand; 3 Department of Pharmacology and Physiology, Faculty of Pharmaceutical Sciences, Chulalongkorn University, Bangkok, 10330 Thailand; 4 Cell-based Drug and Health Product Development Research Unit, Faculty of Pharmaceutical Sciences, Chulalongkorn University, Bangkok, 10330 Thailand; 5 Division of Medical Oncology, Department of Medicine, Faculty of Medicine, Chulalongkorn University, Bangkok, 10330 Thailand

**Keywords:** Non-small-cell lung cancer, Ubiquitylation

## Abstract

The posttranslational modifications (PTMs) of microtubules have been reported to play an important role in cancer aggressiveness, including apoptosis resistance. In this study, we aimed to investigate the biological role of microtubule PTMs in the regulation of paclitaxel responsiveness. The acetylated tubulin (Ace-tub) level was strongly associated with paclitaxel sensitivity, as observed in patient-derived primary lung cancer cells and xenografted immunodeficient mice. We showed that paclitaxel-resistant H460 lung cancer cells, generated by a stepwise increase in paclitaxel, exhibited markedly increased tubulin acetylation and consequently acquired paclitaxel resistance. Upregulation of tubulin acetylation by overexpression of α-tubulin acetyltransferase 1 wild-type (αTAT1^wt^), an enzyme required for acetylation, or by treatment with trichostatin A (TSA), a histone deacetylase 6 (HDAC6) inhibitor, significantly attenuated paclitaxel-induced apoptosis. Investigation of the underlying mechanism revealed that the levels of antiapoptotic Mcl-1 appeared to increase in αTAT1^wt^-overexpressing and TSA-treated cells compared to control cells, whereas the levels of other antiapoptotic regulatory proteins were unchanged. On the other hand, decreased tubulin acetylation by αTAT1 RNA interference downregulated Mcl-1 expression in patient-derived primary lung cancer and paclitaxel-resistant lung cancer cells. A microtubule sedimentation assay demonstrated that Mcl-1 binds to microtubules preferentially at Ace-type, which prolongs the Mcl-1 half-life (T_1/2_). Furthermore, immunoprecipitation analysis revealed that polyubiquitination of Mcl-1 was extensively decreased in response to TSA treatment. These data indicate that tubulin acetylation enhances the resistance to paclitaxel-induced cell death by stabilizing Mcl-1 and protecting it from ubiquitin–proteasome-mediated degradation.

## Introduction

Lung cancer is an aggressive malignancy being the highest causes of cancer-related mortality^1^. The 5-year survival rate of lung cancer patients has considerably declined to only to 19%, even though the therapeutic interventions have greatly improved. Most of lung cancer patients acquire chemotherapeutic resistance, an important hallmark of cancer, and some exhibited intrinsic deregulation of cell death mechanisms^2^. Paclitaxel is a potent anticancer drug that binds to microtubules and inhibits microtubule depolarization during mitosis^3^. Paclitaxel is a first-line treatment for late-stage lung cancer that exhibits high efficacy^4,5^; however, the clinical response rate remains unsatisfactory^6,7^, as chemotherapeutic resistance limits its effective clinical use. A better understanding of the molecular basis of apoptotic deregulation is essential.

Several mechanisms of resistance to paclitaxel-induced apoptosis have been reported, and the significant role of microtubule physiology has been emphasized. Microtubules, which are important cytoskeletal proteins, govern numerous cellular activities, including cell division and cell migration. The altered patterns of microtubule posttranslational modifications (PTMs) and tubulin isotypes in cancer cells play critical roles in mediating aggressive behaviors in cancer through the intracellular trafficking of not only cytoplasmic components but also diverse signaling molecules^8,9^. Tubulin acetylation, a stable form of microtubule PTM in which α-tubulin is acetylated, is increased in breast cancer, which promotes the formation of microtentacles, tubulin**-**based membrane protrusions that facilitate breast cancer cell migration^10,11^. Tubulin acetylation preserves the active status of protein kinase B/Akt, a core component of a key oncogenic signaling pathway, and consequently enhances epithelial-to-mesenchymal transition in lung cancer^12^. Mutation at lysine 40 on α-tubulin, which interferes with tubulin acetylation, conferred resistance to paclitaxel-mediated suppression of cancer cell growth^13^. A recent study also demonstrated that an increase in acetylated microtubules suppresses the activity of caspase-3, an executioner enzyme in apoptosis and that this process is mediated by apolipoprotein C-I pseudogene 1^14^. In addition, a clinical investigation reported that mutation of the β-tubulin gene is tightly associated with a low paclitaxel response rate^8^, and overexpression of βIII-tubulin enhances paclitaxel resistance and disease progression by altering microtubule instability^15^. Furthermore, an increase in the level of βII- and βIII-tubulin, coexisting with acetylated tubulin (Ace-tub), contributes to paclitaxel resistance in ovarian cancer cells^16^. The modulation of tubulin isotype composition on paclitaxel sensitivity is well characterized; however, the potential effect of microtubule PTMs on paclitaxel resistance as well as the molecular signaling involved are not yet fully understood.

In this study, we aimed to investigate the biological roles of microtubule PTMs in paclitaxel sensitivity in lung cancer cells. Our results showed that Ace-tub exhibited a strong correlation with paclitaxel responsiveness. Multiple administrations of paclitaxel-mediated drug resistance by enhancing tubulin acetylation. Genetic manipulation and chemical inhibitors were applied to increase tubulin acetylation, which upregulated the antiapoptotic Mcl-1 level by preventing its degradation through the ubiquitin–proteasome pathway. This study, therefore, provides intriguing evidence that microtubule PTMs confer paclitaxel resistance by altering acetylation and targeting to the antiapoptotic Mcl-1 protein in lung cancer cells.

## Results

### Tubulin acetylation levels are related to paclitaxel sensitivity in patient-derived primary lung cancer cells

**Fig. 1:**
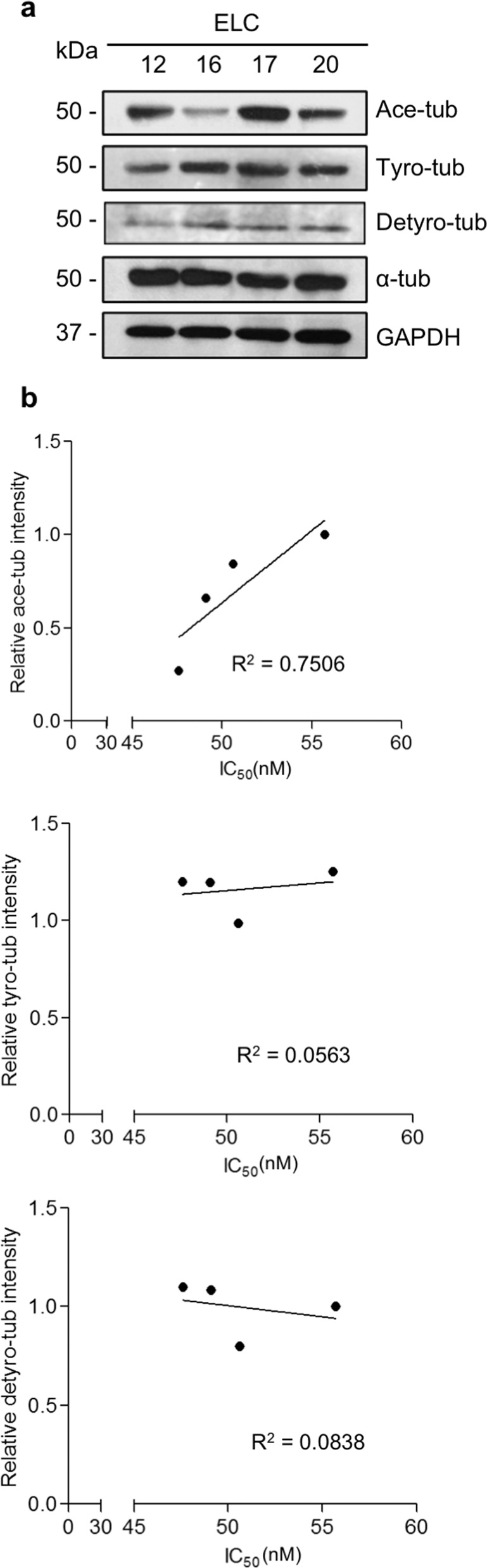
Paclitaxel responsiveness and microtubule posttranslational modification (PTM) in patient-derived primary lung cancer cells. **a** Western blot analysis of acetylated tubulin (Ace-tub), tyrosinated tubulin (Tyro-tub), detyrosinated tubulin (Detyro-tub), and alpha-tubulin (α-tub) expressions in each cell type. Representative blots from three independent experiments are shown. **b** Cells were treated with paclitaxel (0–200 nM) for 48 h, and cell viability was analyzed by MTT assay, and the 50% inhibitory concentration (IC_50_) was assessed (*n* = 3). Plots represent a correlation analysis performed between the relative expression levels of Ace-tub, Tyro-tub, or Detyro-tub and the IC_50_ of paclitaxel in each cell type.

We first investigated the correlation of paclitaxel responsiveness and microtubule PTMs in patient-derived primary lung cancer cells. Analysis of the 50% inhibitory concentration (IC_50_) of paclitaxel demonstrated that ELC17 cells had the highest value (IC_50_ = 55.71 ± 2.13 nM), followed by ELC12 (IC_50_ = 50.60 ± 3.81 nM), ELC20 (IC_50_ = 49.09 ± 3.86 nM), and ELC16 (IC_50_ = 47.59 ± 4.34 nM) cells, respectively. Western blot analysis revealed that the Ace-tub expression level appeared to follow the trend for the IC_50_ value, suggesting that greater Ace-tub expression levels are associated with lower responsiveness to paclitaxel (Fig. 1a). However, tyrosinated tubulin (Tyro-tub) and detyrosinated tubulins (Detyro-tub) levels did not clearly correspond to the IC_50_. To confirm this finding, Pearson’s correlation analysis was performed between the microtubule PTM level and the IC_50_. The results showed that tubulin acetylation exhibited the most relevance to drug sensitivity, with a correlation coefficient of 0.7506, while other PTMs have lower correlation coefficients (Fig. 1b). These data indicated that the level of tubulin acetylation might be related to paclitaxel sensitivity.

### Multiple paclitaxel administrations upregulate tubulin acetylation in an in vivo xenograft

**Fig. 2:**
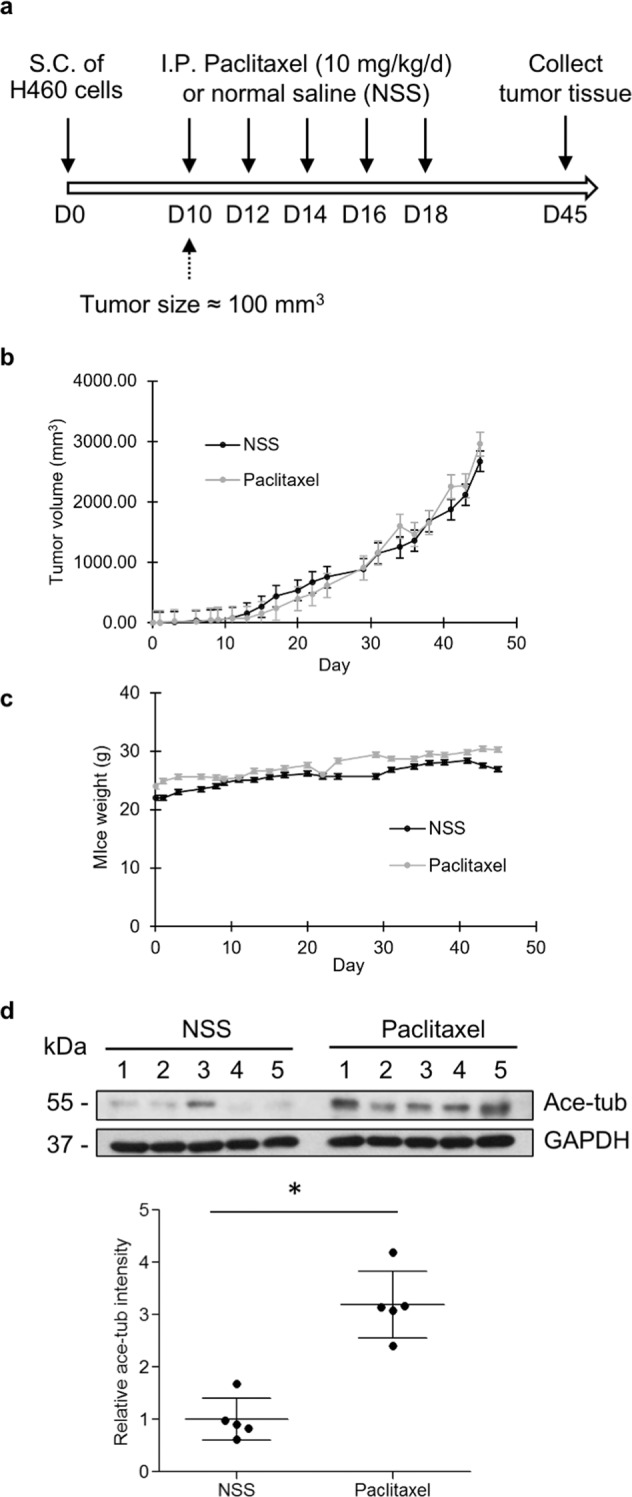
Paclitaxel responsiveness in an in vivo xenograft immunodeficient mouse model. **a** Scheme demonstrating the time course of drug administration. S.C. subcutaneous injection, I.P. intraperitoneal injection. **b** Tumor volume and **c** mouse weight of the paclitaxel- and normal saline (NSS)-treated groups. Plots represent the mean ± SEM from five mice. **d** After 30 days of paclitaxel administration, tumor nodules from the NSS- and paclitaxel-treated groups were collected, and acetylated tubulin (Ace-tub) expression was examined by western blot analysis. The blots were reprobed with GAPDH to confirm equal loading. The graphs show the mean ± SEM of the relative Ace-tub intensity. Student’s *t* test, *P < 0.05 vs NSS-treated group (*n* = 5).

We investigated in vivo paclitaxel responsiveness in xenografted immunodeficient mice. The mice were subcutaneously injected with H460 non-small-cell lung cancer (NSCLC) cells and then treated with drug or normal saline every 2 days for five cycles (Fig. 2a). Tumor nodules were further monitored for 30 days and collected at the end of the experiment. The results showed that during the first period of time (days 10–30), paclitaxel slightly suppressed in vivo tumor growth (Fig. 2b), but after 30 days posttreatment, the tumor nodules in the treatment group gradually expanded, and the size was comparable to that in the control group. However, the mouse weight was not significantly altered from that of the control (Fig. 2c). Interestingly, tubulin acetylation was extensively upregulated in the paclitaxel-treated group (Fig. 2d), suggesting that repetitive paclitaxel administration increased tubulin acetylation levels and might be involved in paclitaxel resistance.

### Tubulin acetylation is upregulated in paclitaxel-resistant lung cancer cells

**Fig. 3:**
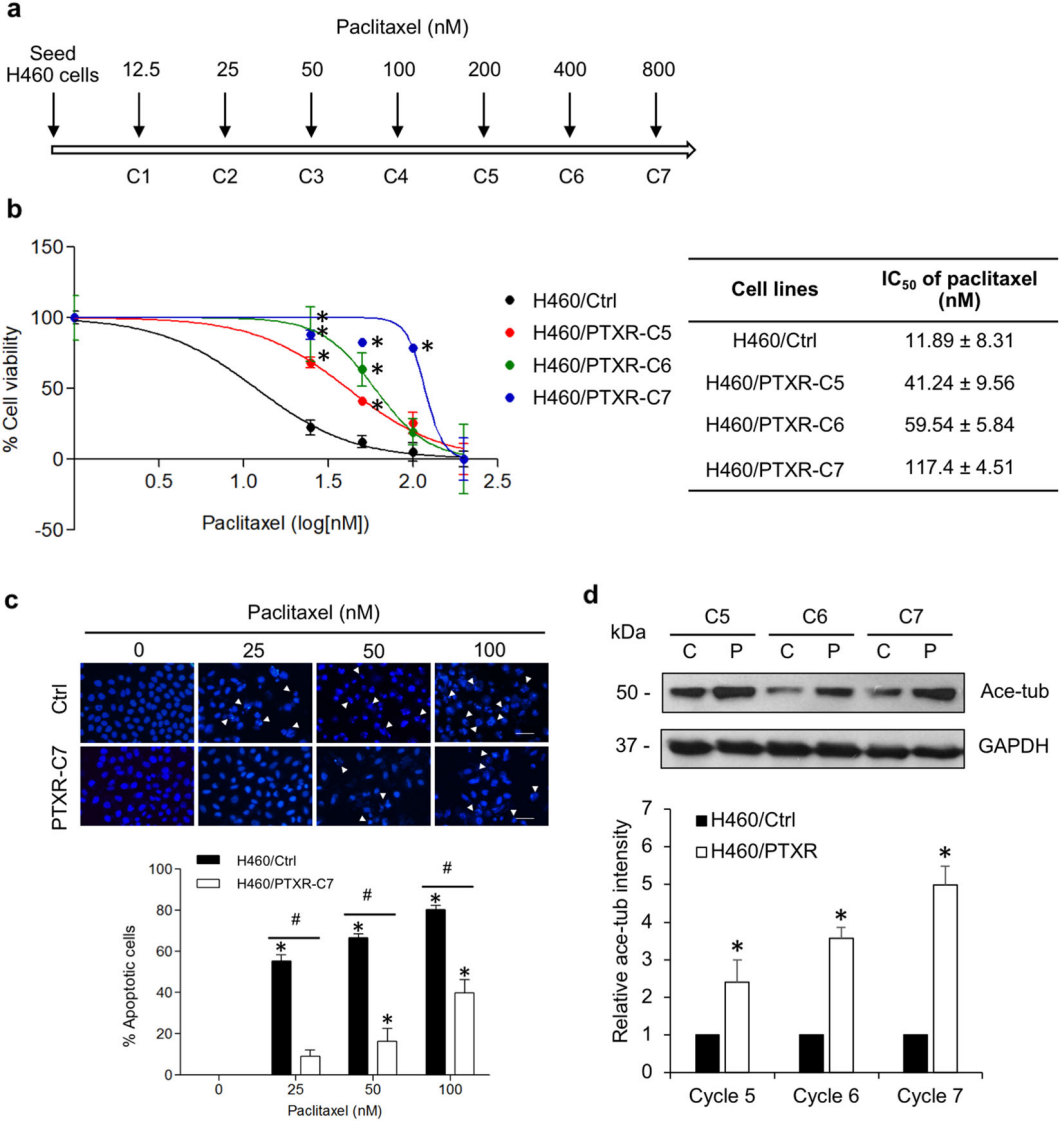
Tubulin acetylation is upregulated in paclitaxel-resistant cells. **a** Scheme demonstrating the time course of paclitaxel-resistant cell establishment. **b** Paclitaxel-resistant H460 (H460/PTXR) cells at cycles 5, 6, and 7 and their passage-matched control H460 (H460/Ctrl) cells were treated with paclitaxel (0–200 nM) for 48 h. Cell viability was analyzed by MTT assay. The graph shows the cell viability (%) as the mean ± SEM (*n* = 3). Student’s *t* test, *P < 0.05 vs H460/Ctrl cells. The table shows the IC_50_ of paclitaxel ± SEM in paclitaxel-resistant (H460/PTXR) and control (H460/Ctrl) cells. **c** Paclitaxel-resistant H460 cells at cycle 7 (H460/PTXR-C7) and control H460 (H460/Ctrl) cells were treated with paclitaxel (0–100 nM) for 48 h. Apoptotic nuclei (white arrowheads) were imaged by fluorescence microscopy. Scale bars: 50 μm. The graph represents the percentage of apoptotic cells as the mean ± SEM (*n* = 3). Student’s *t* test, *P < 0.05 vs nontreated cells; ^#^*P* < 0.05 vs passage-matched control H460 (H460/Ctrl) cells. **d** Acetylated tubulin (Ace-tub) expression in paclitaxel-resistant (H460/PTRX, P) and control (H460/Ctrl, C) cells was examined by western blot analysis. The blots were reprobed with GAPDH to confirm equal loading. The graph shows the mean ± SEM of the relative Ace-tub intensity. Student’s *t* test, *P < 0.05 vs passage-matched control H460 (H460/Ctrl) cells (*n* = 3).

To investigate whether lung cancer cells acquired paclitaxel resistance by upregulation of tubulin acetylation, paclitaxel-resistant H460 NSCLC cells were established by a stepwise increase in paclitaxel concentration from 12.5 nM (cycle 1) to 800 nM (cycle 7) (Fig. 3a), and cell viability in response to paclitaxel was examined. The data revealed that multiple increasing doses of paclitaxel significantly enhanced drug resistance in H460 NSCLC cells (Fig. 3b). The IC_50_ was markedly increased from cycle 1 to 7, cells at cycle 7 (H460/PTXR-C7) treated with paclitaxel up to 800 nM had an IC_50_ ~100-fold greater than that of control cells. Consistently, the number of apoptotic cells with DNA condensation and apoptotic bodies was strikingly decreased in H460/PTXR-C7 cells treated with paclitaxel (Fig. 3c). A dose of 100 nM paclitaxel induced the apoptosis of ~75% of H460/Ctrl cells, whereas ~40% of H460/PTXR-C7 cells underwent apoptosis. Western blot analysis demonstrated that tubulin acetylation was gradually upregulated in paclitaxel-resistant cells compared to their passage-matched controls (Fig. 3d). These results indicate that repeated paclitaxel treatment mediates drug resistance and increases tubulin acetylation levels in NSCLC and that upregulation of tubulin acetylation might be involved in resistance to this drug.

### The overexpression of Ace-tub, triggered by α-tubulin N-acetyltransferase 1 (αTAT1) transfection and histone deacetylase 6 (HDAC6) inhibitor treatment, mediates paclitaxel resistance

**Fig. 4:**
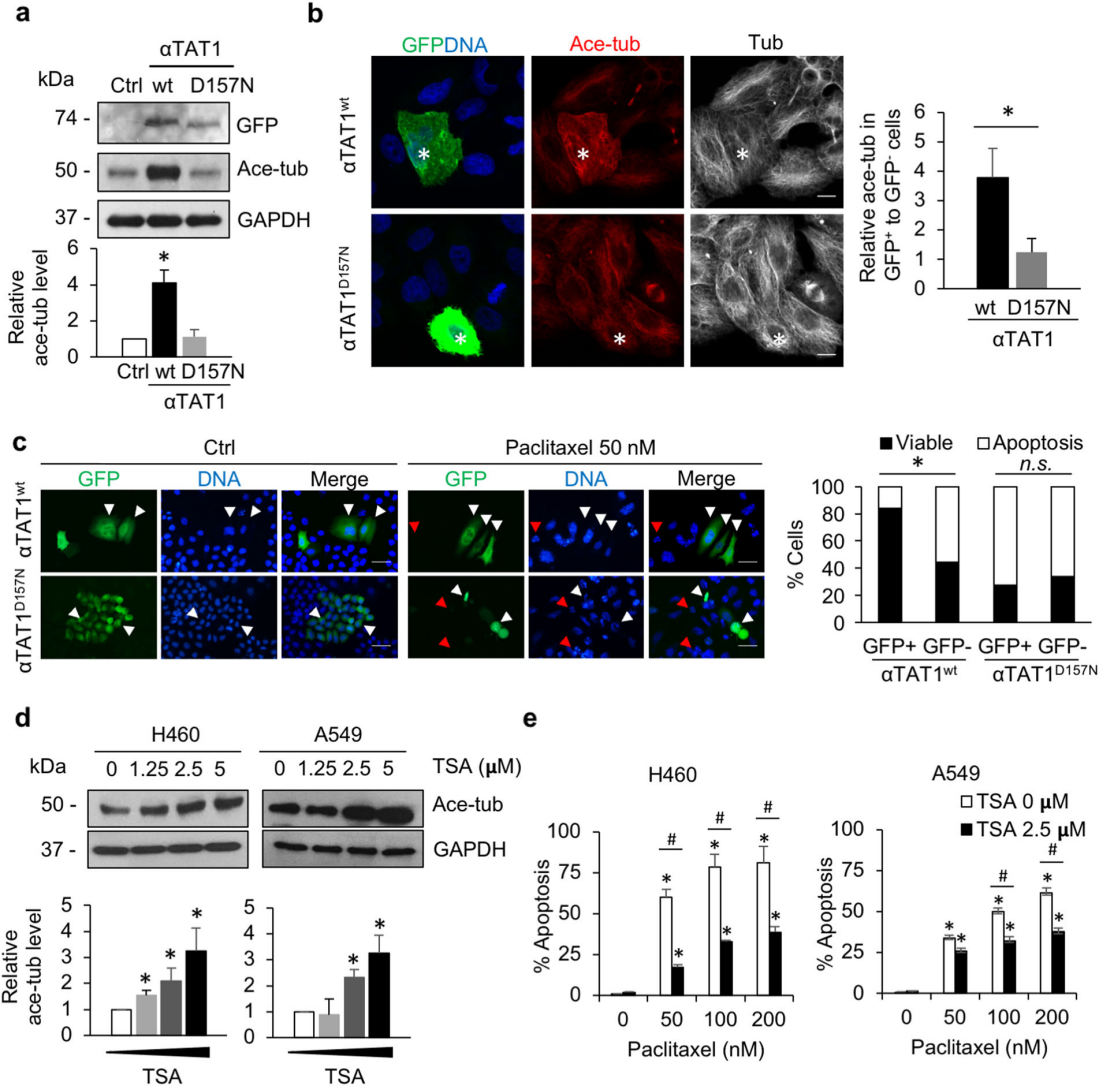
Overexpression of tubulin acetylation mediates apoptotic resistance to paclitaxel. **a** H460 cells were transfected with either GFP-tagged α-tubulin N-acetyltransferase 1 wild-type (GFP-αTAT1^wt^) or its dominant-negative mutant (GFP-αTAT1^D157N^) plasmid. GFP and tubulin acetylation expression was examined by western blot analysis. The blots were reprobed with GAPDH to confirm equal loading. The graph shows the mean ± SEM of the relative acetylated tubulin (Ace-tub) intensity. Student’s *t* test, *P < 0.05 vs mock control H460 (H460/Ctrl) cells (*n* = 3). **b** Cells were immunostained for GFP (green), DNA (blue), acetylated tubulin (Ace-tub; red), and tubulin (gray), and imaged by confocal microscopy. The plot shows the ratio of acetylated tubulin intensity in GFP-positive (asterisk) to GFP-negative cells. Student’s *t* test, *P < 0.05 vs H460/GFP-αTAT1^wt^ cells, *n* = 25 cells. Scale bars: 10 μm. **c** GFP-tagged α-tubulin N-acetyltransferase 1 wild-type (GFP-αTAT1^wt^) or its negative mutant (GFP-αTAT1^D157N^)-transfected H460 cells were treated with 50 nM paclitaxel for 48 h, and GFP and apoptotic nuclei were imaged by fluorescence microscopy. White arrowheads indicate the nuclei of GFP-positive cells, whereas the red arrowhead indicates those of GFP-negative cells. Scale bars: 50 μm. The graph shows the percentage of apoptotic and viable cells in response to paclitaxel. Student’s *t* test, *P < 0.05 vs GFP-positive transfectants. n.s. not significant. **d** H460 and A549 cells were treated with trichostatin A (TSA) for 4 h, and tubulin acetylation levels were examined by western blot analysis. The blots were reprobed with GAPDH to confirm equal loading. The graph shows the mean ± SEM of the relative acetylated tubulin (Ace-tub) intensity. Student’s *t* test, *P < 0.05 vs nontreated cells (*n* = 3). **e** H460 and A549 cells were pretreated with or without TSA (2.5 μM) for 4 h, and further incubated with paclitaxel (0–200 nM) for 48 h. Apoptotic cells were examined by Hoechst 33342 staining and visualized under a fluorescence microscope. The graph shows the percentage of apoptotic cells ± SEM (*n* = 3). Student’s *t* test, *P < 0.05 vs paclitaxel nontreated cells. ^#^*P* < 0.05 vs TSA nontreated cells.

αTAT1 is a primary enzyme responsible for the addition of an acetyl group on α-tubulin at lysine 40^12^; thus, to verify the role of tubulin acetylation on paclitaxel sensitivity, tubulin acetylation was induced by transfection with GFP-tagged αTAT1 wild-type (GFP-αTAT1^wt^) or its dominant-negative mutant (GFP-αTAT1^D157N^) plasmid, whose enzymatic activity was suppressed. Western blot analysis demonstrated the corresponding GFP expression in these transfectants. Tubulin acetylation was upregulated in GFP-αTAT1^wt^-transfected H460 cells (H460/αTAT1^wt^), whereas it was not altered in H460/αTAT1^D157N^ cells compared to mock-transfected control cells (H460/Ctrl) (Fig. 4a). Immunofluorescence assays also revealed that the ratio of acetylated microtubules in the GFP-positive group was greater than that in the GFP-negative group of H460/αTAT1^wt^ cells, whereas no significant change was observed between the GFP-positive and -negative cells of the H460/αTAT1^D157N^ group (Fig. 4b). Because the transfection efficiency was very low, apoptotic cell death was closely observed at an individual cell level. The data showed that apoptotic cell death mediated by paclitaxel was clearly attenuated in GFP-positive H460/αTAT1^wt^ cells, whose tubulin acetylation was enhanced, compared to GFP-negative H460/αTAT1^wt^ cells (Fig. 4c). However, the apoptosis rate of GFP-positive H460/αTAT1^D157N^ cells was comparable to that of GFP-negative cells.

To confirm the above finding, H460 and A549 NSCLC cells were treated with various noncytotoxic concentrations of trichostatin A (TSA), an inhibitor of HDAC6, which is a dominant enzyme required for the removal of the acetyl group from α-tubulin, to minimize the interference of this factor in cell death (Supplementary Fig. 1). Western blot analysis showed that tubulin acetylation was gradually upregulated in a dose-dependent manner (Fig. 4d). Furthermore, paclitaxel-mediated apoptosis was attenuated by TSA treatment. The apoptosis rate was ~80% among H460 cells treated with paclitaxel (200 nM), whereas it was reduced to 45% in the presence of TSA (Fig. 4e, left). Consistently, the apoptosis rate of A549 cells was significantly lower in the TSA-pretreated group than in the group treated with paclitaxel alone (Fig. 4e, right). These data indicate the important role of tubulin acetylation in the regulation of paclitaxel responsiveness.

### Tubulin acetylation upregulates the levels of antiapoptotic Mcl-1

**Fig. 5:**
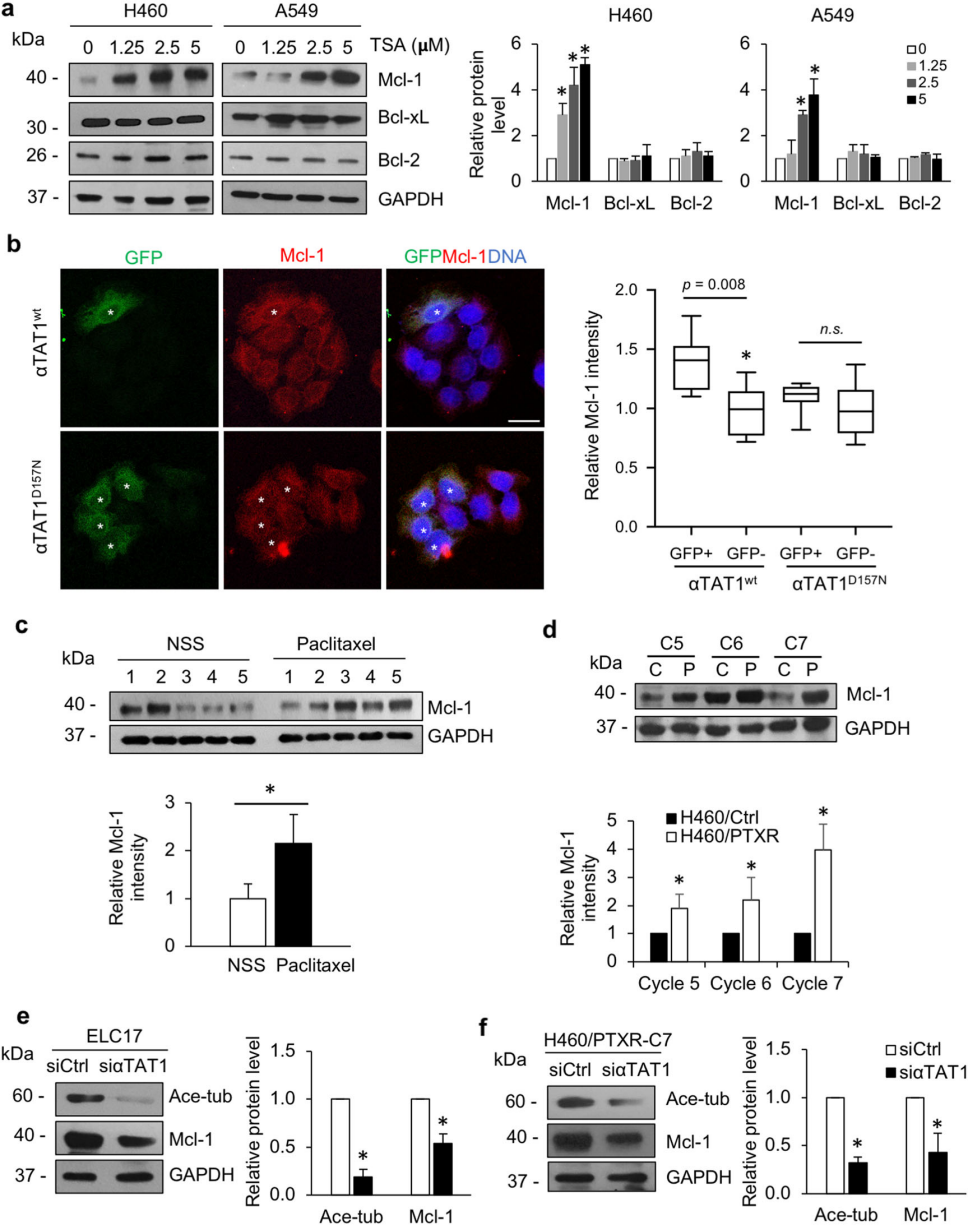
Tubulin acetylation upregulates antiapoptotic Mcl-1 level. **a** H460 and A549 cells were treated with various concentrations of TSA for 4 h, Mcl-1, Bcl-xL, and Bcl-2 expressions were examined by western blot analysis. The blots were reprobed with GAPDH to confirm equal loading. The graph shows the mean ± SEM of the relative protein expression level. Student’s *t* test, *P < 0.05 vs nontreated cells (*n* = 3). **b** H460 cells were transfected with either GFP-tagged α-tubulin N-acetyltransferase 1 wild-type (GFP-αTAT1^wt^) or its dominant-negative mutant (GFP-αTAT1^D157N^) plasmid, and immunostained for GFP (green), Mcl-1 (red), and DNA (blue). Cells were imaged by confocal microscopy. The plot shows the relative intensity of Mcl-1 in GFP-positive (asterisk) and -negative cells. Data were analyzed from GFP-αTAT1^wt^ cells (*n* = 35) and GFP-αTAT1^D157N^ cells (*n* = 56). Scale bars: 20 μm. n.s. not significant. **c** After 30 days of paclitaxel administration, tumor nodules from normal saline (NSS)- and paclitaxel-treated groups were collected, and Mcl-1 expression was examined by western blot analysis. The blots were reprobed with GAPDH to confirm equal loading. The graph shows the mean ± SEM of the relative Mcl-1 intensity. Student’s *t* test, *P < 0.05 vs normal saline (NSS)-treated group (*n* = 5). **d** Mcl-1 expressions in paclitaxel-resistant (H460/PTRX, P) and their control (H460/Ctrl, C) cells were examined by western blot analysis. The blots were reprobed with GAPDH to confirm equal loading. The graph shows the mean ± SEM of the relative Mcl-1 intensity. Student’s *t* test, *P < 0.05 vs passage-matched control H460 (H460/Ctrl; C) cells (*n* = 3). **e** Paclitaxel-resistant (H460/PTRX-C7) and **f** patient-derived primary lung cancer ELC17 cells were transfected with either siRNA to αTAT1 or control, acetylated tubulin and Mcl-1 levels were examined by western blot analysis. The blots were reprobed with GAPDH to confirm equal loading. The graph shows the mean ± SEM of the relative protein intensity. Student’s *t* test, *P < 0.05 vs siCtrl cells (*n* = 3).

To gain insight into the molecular mechanism, the antiapoptotic regulatory proteins in the Bcl-2 family were investigated. H460 and A549 NSCLC cells were treated with TSA, and protein expression was examined by western blot analysis. The data showed that antiapoptotic Mcl-1 was extensively elevated in a dose-dependent manner, whereas the other antiapoptotic proteins, Bcl-xL and Bcl-2, were unchanged in both H460 and A549 NSCLC cells (Fig. 5a); the changes in Mcl-1 levels corresponded to the changes in tubulin acetylation levels (Fig. 4d). Consistently, immunofluorescence assays demonstrated that GFP-αTAT1^wt^-overexpressing H460 cells (H460/GFP-αTAT1^wt^) exhibited an increase in Mcl-1 levels compared to those in GFP-negative cells, whereas the Mcl-1 levels were not altered in H460/GFP-αTAT1^D157N^ cells (Fig. 5b). Interestingly, an in vivo xenograft experiment revealed that upregulation of Mcl-1 was clearly observed in the paclitaxel-treated group (Fig. 5c). Furthermore, paclitaxel-resistant cells exhibited elevated Mcl-1 expression compared to that in their passage-matched controls (Fig. 5d), indicating an association of Mcl-1 expression and tubulin acetylation levels.

To test the hypothesis that tubulin acetylation regulates Mcl-1 levels, ELC17, and H460/PTXR-C7 cells, which have the highest tubulin acetylation levels among the cells assessed, were transfected with siRNA targeting αTAT1. Western blot analysis revealed that tubulin acetylation was decreased in response to αTAT1 knockdown in both ELC17 cells (Fig. 5e) and H460/PTXR-C7 cells (Fig. 5f). Furthermore, Mcl-1 expression was significantly decreased in siαTAT1-transfected cells, suggesting that tubulin acetylation positively regulates Mcl-1 expression.

### Tubulin acetylation stabilizes the antiapoptotic protein Mcl-1 and prevents its ubiquitin–proteasome-mediated degradation

**Fig. 6:**
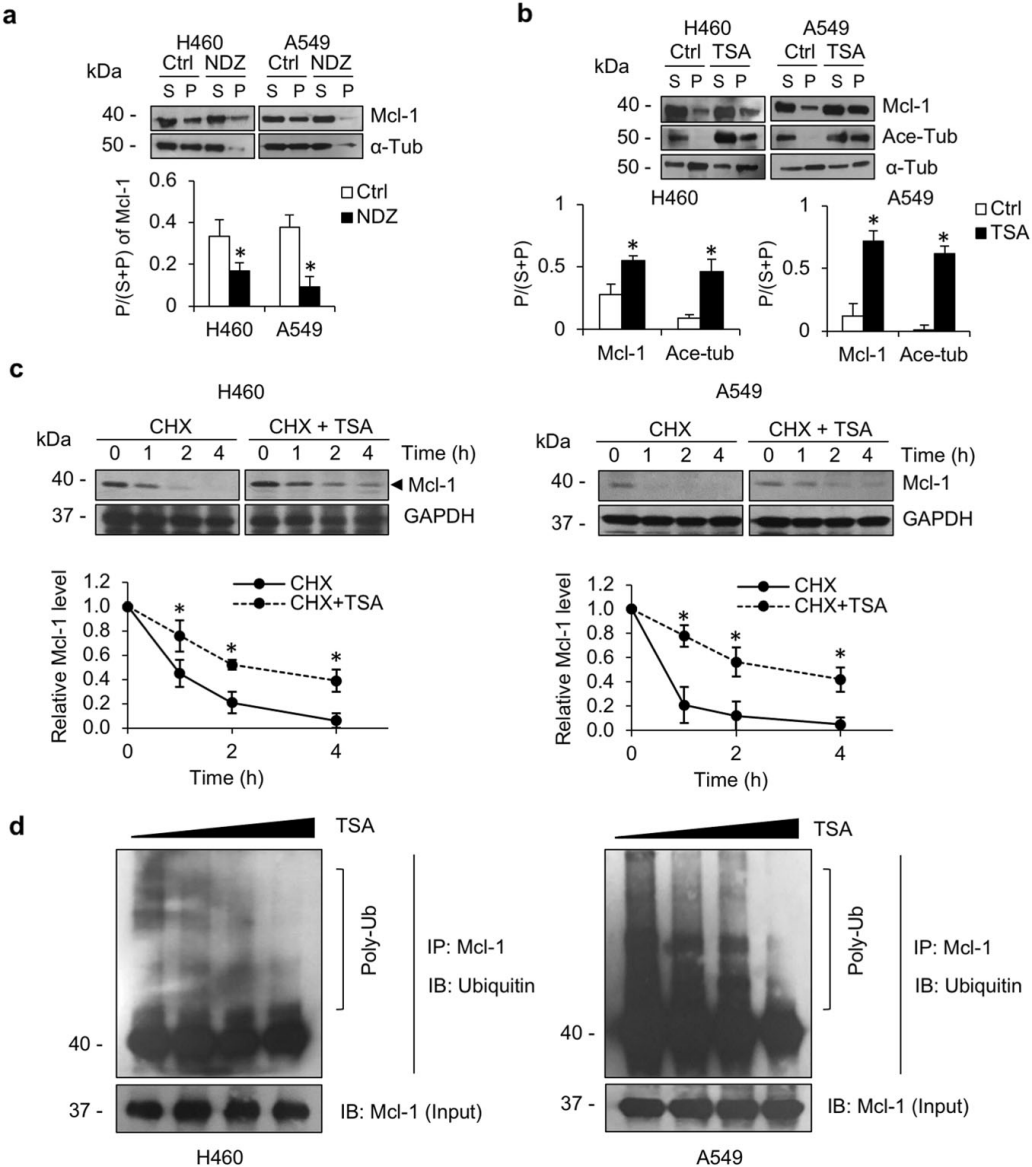
Tubulin acetylation stabilizes Mcl-1 and protects it from degradation-mediated by the ubiquitin–proteasome pathway. **a** H460 and A549 cells were treated with or without 10 μM nocodazole (NDZ) for 1 h. Lysates were separated into pellet (P) and soluble (S) fractions by microtubule sedimentation assay, and analyzed for Mcl-1 and α-tubulin (α-tub) by western blot analysis. The plot shows the ratio of Mcl-1 in the pellet (P) to the total fraction (S + P) as the mean ± SEM. Student’s *t* test, *P < 0.05 vs control group (*n* = 3). **b** H460 and A549 cells were treated with or without 5 μM tricostatin A (TSA) for 4 h. Lysates were separated into pellet (P) and soluble (S) fractions by microtubule sedimentation assay, and analyzed for Mcl-1, acetylated tubulin (Ace-tub), and α-tubulin (α-tub) by western blot analysis. The plot shows the ratio of Mcl-1 in the pellet (P) to the total fraction (S + P) as the mean ± SEM. Student’s *t* test, *P < 0.05 vs control group (*n* = 3). **c** H460 and A549 cells were treated with cycloheximide (CHX, 10 μg/mL) in the presence or absence of TSA (5 μM) for 0-4 h. Mcl-1 expression was examined by western blot analysis. The blots were reprobed with GAPDH to confirm equal loading. The graph shows the mean ± SEM of the relative Mcl-1 intensity. Student’s *t* test, *P < 0.05 vs CHX-treated cells at each time point (*n* = 3). **d** H460 and A549 cells were treated with MG132, a proteasomal inhibitor with or without TSA (5 μM) for 4 h. Polyubiquitination (Poly-Ub) of Mcl-1 was analyzed by immunoprecipitation with an antibody to Mcl-1 and immunoblotted with antibody to ubiquitin. Represented blots from three independent experiments are shown.

Since microtubules have been reported to be involved in cellular trafficking and to regulate several molecules interacted^17^, we hypothesize that microtubule PTMs, especially acetylation, might modulate Mcl-1 binding on them. The interaction of Mcl-1 with microtubules was first investigated by a microtubule sedimentation assay. The results revealed that Mcl-1 was observed in both the pellet fraction (P) of microtubules and the supernatant fraction (S) of other intracellular compartments (Fig. 6a). However, after treatment with nocodazole, a microtubule-disrupting agent, Mcl-1 in the microtubule fraction (P) was clearly diminished, and the ratio of Mcl-1 in the pellet to the total fraction in both cell lines was significantly decreased, indicating that Mcl-1 adhered to the microtubules. Furthermore, the accumulation of Mcl-1 in the microtubule compartment (P) was notably increased in the presence of TSA, a deacetylation inhibitor (Fig. 6b), indicating that Mcl-1 localized preferentially to microtubule acetylation.

The ubiquitin–proteasome-mediated degradation process is reported to regulate intracellular Mcl-1 levels^18,19^. The degradation rate of Mcl-1 was examined by pretreatment of the cells with cycloheximide (CHX), a protein synthesis inhibitor, and the endogenous Mcl-1 levels were investigated. Western blot analysis revealed that Mcl-1 levels were declined over time, with T_1/2_ values of 0.89 ± 0.11 h and 0.35 ± 0.16 h in H460 and A549 cells, respectively (Fig. 6c). However, in the presence of TSA, the Mcl-1 level was stable, and the T_1/2_ appeared to increase to 2.35 ± 0.25 h in H460 cells and to 2.99 ± 0.54 h in A549 cells (Fig. 6c). Furthermore, to test the hypothesis that Ace-tub prevents Mcl-1 degradation via a ubiquitin–proteasome-mediated mechanism, the polyubiquitination of Mcl-1 was investigated. Immunoprecipitation assay demonstrated that the polyubiquitination of Mcl-1 was notably suppressed in response to TSA treatment in a dose-dependent manner (Fig. 6d). This result indicates that tubulin acetylation is required for Mcl-1 stability, contributing to paclitaxel resistance.

## Discussion

Apoptosis resistance remains a serious clinical problem, especially in lung cancer^20^. Although its clinical management has advanced, resistance to chemotherapeutic drugs often occurs, leading to undesirable outcomes. Paclitaxel, a standard therapy in lung cancer, exhibits an effective curative rate; however, the acquisition of drug resistance and cancer relapse appears to be major obstacles^21^. A better understanding of the underlying molecular mechanism could provide a therapeutic strategy or drug target for achieving satisfactory outcomes in the clinical. In the current work, we showed for the first time on the mechanism of resistance to paclitaxel-mediated apoptosis, which is regulated by microtubule PTMs in NSCLCs. Multiple administrations of paclitaxel enhanced tubulin acetylation and consequently upregulated Mcl-1 levels. We further found that Mcl-1 interacts with microtubules, preferentially on Ace-tub, which is important for Mcl-1 stabilization, protecting it from degradation and mediating drug resistance.

Microtubules, important intracellular cytoskeletal components, are well known as key players in maintaining of cellular morphology and supporting several cellular activities. The hollow-like structure of microtubules consisted of α- and β-tubulin dimers, and the elongation and shrinkage of tubulin dimers enable their dynamic nature and thus their cellular functions^22^. Their regulatory roles, in association with oncogenic functions, are often emphasized in cancer cell biology and are targets for various anticancer drugs^8,23^. Paclitaxel, a microtubule-stabilizing drug, directly binds to β-tubulin and inhibits microtubule disassembly during cell division, consequently mediating cell death^24^. Accumulating studies have demonstrated that prolonged treatment with paclitaxel leads to drug resistance in an association with tubulin-related changes, including alterations in tubulin isotypes and microtubule PTMs. Clinical investigations have reported that patients with mutations in β-tubulin have a low response rate to such drugs^15^ and that upregulation of class III β-tubulin leads to resistance to paclitaxel in ovarian cancer^25–27^. Likewise, microtubule PTMs, paclitaxel-resistant breast cancer cells exhibit an elevation in Tyro-tub^28^ and an increase in polyglutamylated microtubules in cooperation with septin filaments^29^. We also found a strong correlation of α-tubulin acetylation with paclitaxel sensitivity in patient-derived primary lung cancer cells (Fig. 1). Upregulation of tubulin acetylation was observed in xenograft tissues after the complete course of drug administration (Fig. 2d), but tumor size was not affected. Consistently, paclitaxel-resistant cells, established by treatment with increasing concentrations of this drug, exhibited markedly elevated microtubule acetylation and resistance to cell death (Fig. 3), indicating the possible role of α-tubulin acetylation in apoptosis evasion. Since paclitaxel has been reported to stabilize polymerized microtubules, in which tubulins are marked by acetylation^30^, hyperacetylation of tubulin mediated by long-term treatment with paclitaxel might conversely cause aberrant cell death signaling rather than apoptosis induction^31,32^.

Accumulating studies have provided evidence that paclitaxel resistance is mediated by the dysregulation of apoptotic signaling pathways, including apoptotic regulatory proteins in the Bcl-2 family^33^. Upregulation of antiapoptotic Bcl-2, Mcl-1, and Bcl-xL was found in paclitaxel-resistant esophageal cell lines and xenograft tissues^34^. The restoration of Bcl-2 family proteins, either by suppression of antiapoptotic proteins or by induction of proapoptotic proteins, was shown to confer paclitaxel resistance^35,36^. Here, we demonstrated that Mcl-1 levels appeared to increase in paclitaxel-resistant lung cancer cells and in the tumor nodules of mice who completed the paclitaxel treatment cycles (Fig. 5c, d). Since tubulin alterations are closely associated with paclitaxel sensitivity, there is less conclusive evidence demonstrating the interplay of microtubules in the regulation of Bcl-2 family proteins. Notably, microtubule filaments play an important role in the intracellular translocation of organelles and molecules, preserving their cargo activities^37^. Recently, we reported that tubulin acetylation of microtubules maintains Akt activity, and promotes epithelial-to-mesenchymal transition in lung cancer cells^12^. The trafficking of vesicles containing focal adhesion components to the leading edge of migrating cells requires microtubule acetylation^38^. In addition, microtubules act as scaffolds for Hsp90, which is necessary for the stimulation of apoptosis and cell proliferation-related targets^39^. In this study, we underscore the important role of microtubules in regulating the antiapoptotic protein Mcl-1. Overexpression of αTAT1, but not the negative mutant, induced upregulation of tubulin acetylation that led to an increase in Mcl-1 and resistance to paclitaxel-mediated apoptosis (Figs. 4c and 5b), indicating that microtubule acetylation is essential for Mcl-1 expression, facilitating the apoptosis avoidance mechanism of this drug.

**Fig. 7:**
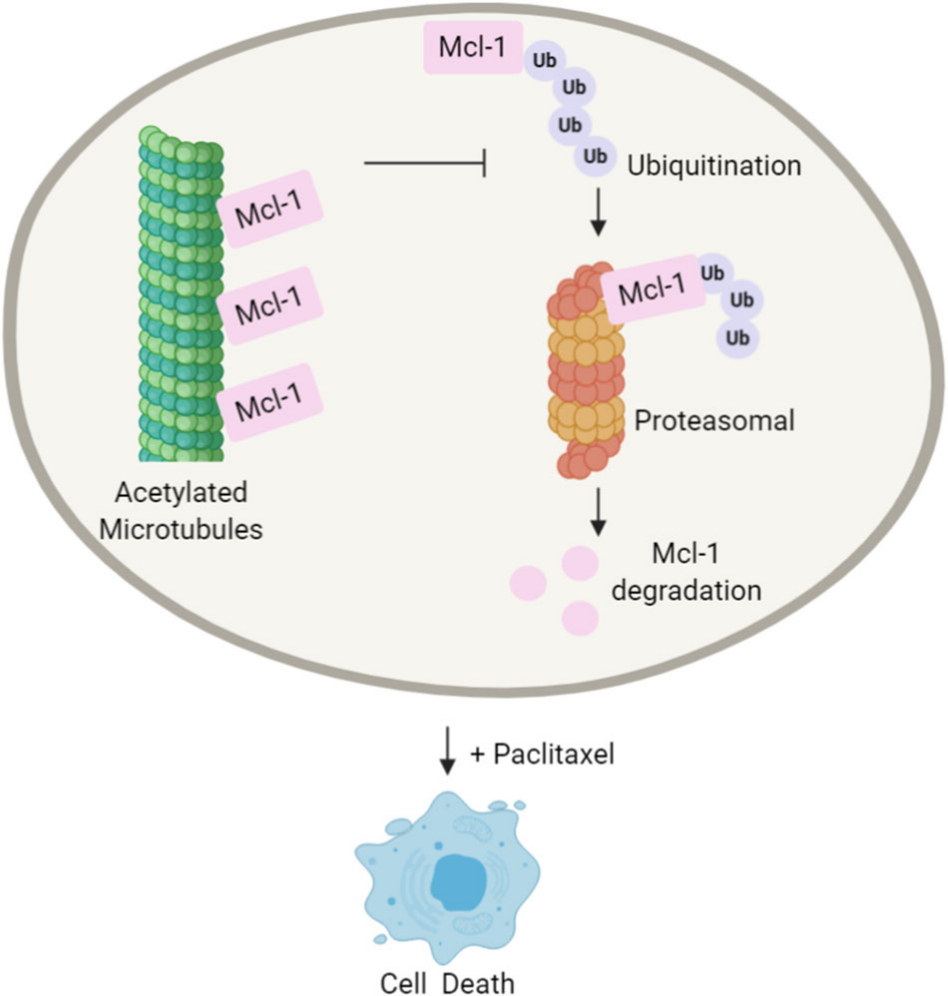
Schematic presentation showing the regulation of tubulin acetylation on antiapoptotic Mcl-1 and paclitaxel resistance. Multiple administrations of paclitaxel induce upregulation of tubulin acetylation and Mcl-1 level. Mcl-1 preferentially interacts with this posttranslational modification (PTM) of microtubules, in which tubulin acetylation functions to stabilize Mcl-1 by protecting it from ubiquitin-mediating proteasomal degradation, and thereby conferring resistance to paclitaxel-mediated apoptosis.

It is well known that the ubiquitin–proteasome pathway is a dominant pathway for protein degradation that involves with apoptotic regulatory proteins^40^. Substrates degraded via this pathway are chemically ubiquitinated prior to binding with and be degraded by the proteasome. A previous study reported that the intracellular level of Mcl-1 was mainly determined by its degradation mechanism through the proteasomal pathway^41–43^. Inhibition of the Mcl-1 degradation process was shown to be related to apoptotic susceptibility to microtubule-targeting drugs^44^. In the present study, we identified a novel connection between microtubules and Mcl-1 degradation, in which tubulin acetylation functions to stabilize Mcl-1 by protecting it from ubiquitin-mediating proteasomal degradation (Fig. 6d). Increasing of the Ace-tub level with the deacetylase inhibitor TSA strikingly increased Mcl-1 levels by suppressing its degradation (Fig. 6c). Taken together, the present findings provide mechanistic insight regarding the role of microtubule PTMs in apoptosis regulation and highlight the linkage of microtubule acetylation to the stability of antiapoptotic Mcl-1, which has a significant impact on paclitaxel responsiveness (Fig. 7). This study indicated the potential role of tubulin acetylation as a predictive marker for paclitaxel susceptibility and a promising target against drug resistance in cancer.

## Materials and methods

### Cell culture and chemicals

Non-small-cell lung cancer (NSCLC) H460 and A549 cells were obtained from American Type Culture Collection (ATCC; Manassas, VA, USA). The patient-derived malignant cancer cells (ELC12, ELC16, ELC17, and ELC20) were isolated from pleural effusions of recurrent or advanced stage non-small-cell lung cancer patients who had been diagnosed at the King Chulalongkorn Memorial Hospital. The protocol of conduction was approved by the Ethics Committee of the Faculty of Medicine, Chulalongkorn University, Bangkok, Thailand (IRB 365/62), and was obtained informed consent from all participants. This study was carried out in an accordance with the principles of the World Medical Association Declaration of Helsinki. All cells were maintained in RPMI supplemented with 10% fetal bovine serum, 1% l-glutamine, and 1% penicillin/streptomycin under 37 °C and 5% CO_2_.

Paclitaxel, 3-(4,5-dimethylthiazol-2-yl)-2,5-diphenyltetrazolium bromide (MTT), dimethyl sulfoxide (DMSO), Hoechst 33342, and DAPI were purchased from Sigma (St Louis, MO, USA). Paclitaxel Injection USP (Intaxel^®^) was obtained from Fresenius Kabi Oncology Co., Ltd. (Solan, India). A 0.9% sodium chloride injection was from Thai Nakorn Patana Co., Ltd. (Bangkok, Thailand). The primary antibodies used were anti-Mcl-1 (Cell Signaling Technology #94296), anti-Bcl-2 (Cell Signaling Technology #4223), anti-Bcl-xL (Cell Signaling Technology #2764), anti-GAPDH (Cell Signaling Technology #97166), anti-tubulin (Sigma #T6199), anti-tyrosinated tubulin (Sigma #MAB1864-I), anti-detyrosinated tubulin (Sigma #AB3201), anti-acetylated tubulin (Sigma #T7451), and anti-ubiquitin (Dako #Z0458). The secondary antibodies used were HRP-conjugated anti-rabbit IgG (Cell Signaling Technology #7074) and anti-mouse IgG (Cell Signaling Technology #7076).

### Establishment of paclitaxel-resistance cells

Paclitaxel-resistance H460 (H460/PTX) cells were established by a stepwise increment with paclitaxel^12^. At each step, cells were maintained in a cell culture medium containing paclitaxel for at least two weeks and subcultured every 2 days. The drug concentration was gradually increased two-fold from 12.5 to 800 nM. Cell viability assay was performed to evaluate drug sensitivity, and at least a 100-fold increase of IC50 comparing with control cells was considered as drug-resistant cells.

### In vivo xenograft tumorigenesis

A 10 of 6-week-old male BALB/cMlac-nu nude mice were purchased from Nomura Siam International (Bangkok, Thailand). Mice were housed with an environmental enrichment maintaining under stringently hygiene conditions with 24 ± 2 °C temperature-controlled, 40–60% humidity, and a 12:12 h light–dark cycle at the Laboratory Animal Center at Faculty of Pharmaceutical Sciences, Chulalongkorn University. The study was approved by the Institutional Animal Care and Use Committee of the Faculty of Pharmaceutical Sciences, Chulalongkorn University, Bangkok, Thailand (CU-AUP 19-33-003). Both flanks of mice were subcutaneously inoculated with 5 × 10^6^ cells of H460 cells. Mice were weighed, and the tumor volume was observed every 2 days using Vernier caliper as following: (length × width × width)/2. After 7 days, mice were randomly divided into two groups (*n* = 5), and 10 mg/kg paclitaxel or 0.9% normal saline was intraperitoneal injection every 2 days for five times. After 45 days, mice were euthanized, and the tumors were dissected and stored at −20 °C for further biochemical assays.

### Cell viability assay

A number of 10^4^ cells/well were cultured onto a 96-well plate overnight. Cells were treated with paclitaxel for 48 h, and then media was replaced by MTT (5 mg/mL) solution and incubated for 3 h. The formazan product was solubilized by DMSO, and its absorbance was measured at 570 nm by a microplate reader (Perkin Elmer VICTOR3/Wallac 1420). Viable cells were calculated and represented as a percentage of cell viability, by which the control group was considered as 100%. The inhibitory concentration at 50% of cell viability was identified using Prism 8 software (GraphPad).

### Apoptosis assay

After treatment with paclitaxel for 48 h, cells were incubated with H33342 (2 μg/mL) at 37 °C for 15 min in the dark. Cells were imaged at least five random field/groups under a fluorescent microscope (Nikon Inverted Microscope Eclipse Ti-U/B, NY, USA). A number of apoptotic cells to total cell number was calculated and presented as percentage of apoptotic cells.

### Plasmids and transfections

GFP-tagged-α tubulin acetyltransferase 1 wild-type (αTAT1-wt) and its dominant-negative, D157N plasmids (αTAT1-DN) were gifted from Professor Takeichi Masatoshi (RIKEN Center for Biosystems Dynamic Research, Japan). Plasmid transfection was performed using Lipofectamine^®^ 2000 (Invitrogen, Waltham, MA, USA) according to the manufacturer’s protocol. Briefly, a 2 μg of plasmid in optiMEM media was mixed with 4 μl of Lipofectamine^®^ 2000 and incubated for 15 min at room temperature. The mixture was drooped onto the cells and incubated further at 37 °C for 6 h. Protein overexpression was examined by either western blot analysis or immunofluorescence assay. Cells were subcultured and seeded for other biochemical assays.

### siRNA and transfections

αTAT1 knockdown was performed using Lipofectamine^®^ RNAiMAX following the manufacturer’s instruction. Stealth RNAi to αTAT1 and control were purchased from Invitrogen (Invitrogen, Waltham, MA, USA). The sequence of siαTAT1 was 5′-ACCGCACCAACTGGCAATTGA-3. Briefly, 100 nM of stealth RNAi targeting to αTAT1 in OptiMEM (Invitrogen) was incubated with Lipofectamine^®^ RNAiMAX in OptiMEM for 15 min at room temperature. The mixture was dropped wisely added onto the cells and incubated further at 37 °C for 6 h. After transfection for 72 h, cells were subjected to western blot analysis.

### Western blot analysis

Cells were lysed with TMEN buffer (20 mM Tris-HCl, pH 7.5; 1 mM MgCl_2_; 150 mM NaCl; 20 mM NaF; 0.5% sodium deoxycholate; 1% nonidet-40; 0.1 mM phenylmethylsulfonyl fluoride; and cOmplete^TM^ protease inhibitor cocktail, Roche) on ice for 40 min. The supernatant was collected by centrifugal at 12,000 × *g* at 4 °C for 20 min. Protein content was measured by BCA Protein Assay Kit (Pierce^TM^, Thermo Fisher Scientific, CA, USA). An equal amount of protein was dissolved in SDS-polyacrylamide gel electrophoresis and transferred to the PVDF membrane. Blots were incubated with 5% skim milk in TBS-T buffer (Tris buffer saline with 0.075% Tween-20) for 30 min at room temperature, specific primary antibody at 4 °C overnight, and correspondent secondary antibody for 2 h at room temperature. An equal of loading was confirmed by GAPDH expression, using anti-GAPDH antibody (Cell Signaling Technology #97166). Protein signals were detected by the chemiluminescence system (Merck Millipore, MA, USA). The relative protein intensity was analyzed and normalized to GAPDH by ImageJ software (NIH).

### Immunofluorescence assay

Cells were seeded onto coverslip in a 24-well plate. After indicated treatment, cells were fixed with cold methanol at −20 °C for 5 min and blocked in 3% bovine serum albumin (BSA) at room temperature for 30 min. Cells were incubated with rabbit anti-GFP antibody (1:500, MBL #598), mouse anti-acetylated tubulin (1:2000, Sigma #T7451) or rat anti-α-tubulin (1:2000, Millipore #MAB1864) at 4 °C overnight, washed with PBS followed by incubation with goat AlexaFluor 488-conjugated anti-rabbit (1:500, Invitrogen #A32731), 568-conjugated anti-mouse (1:1000, Invitrogen #A11031) or 647-conjugated anti-rat IgG (1:000, Invitrogen #A21236) for 2 h at room temperature. After washing with PBS containing DAPI, coverslips were washed with deionized water and mounted with FluorSave (EMD Millipore). Images were acquired by fluorescence microscope (Nikon Inverted Microscope Eclipse Ti-U/B, NY, USA). Fluorescence intensity was quantified by ImageJ software (NIH) as described^12^.

### Microtubule sedimentation assay

The interaction of proteins on microtubule was analyzed as described^12^. Briefly, after indicated treatment, cells were incubated with 1 μM Taxol for 30 min at 37 °C and dissolved in a microtubule-stabilizing buffer containing 80 mM PIPES, 80 mM K-1,4-piperazinediethanesulfonic acid (pH 6.8), 1 mM EGTA, 1 mM MgCl_2_, 0.5% (vol/vol) nonidet P-40, 20 mM NaF, 0.5% sodium deoxycholate, 10 mM Taxol, 0.1 mM phenylmethylsulfonyl fluoride, and cOmplete^TM^ protease inhibitor cocktail (Roche) for 5 min at 37 °C in the dark. The microtubule fraction was separated by centrifugal at 18,000 × *g* for 15 min at 30 °C. The pellet was washed with microtubule-stabilizing buffer without detergent and resuspended with the same buffer in an equal volume of supernatant. Both pellet and supernatant fractions were boiled in a sampling buffer at 95 °C and analyzed by western blot analysis.

### Immunoprecipitation assay

Cells were pretreated with proteasomal inhibitor MG132 (10 μM) for 1 h and incubated with 5 μM trichostatin A (TSA) for indicated time points. Cells were then lysed in TMEM lysis buffer for 45 min on ice. The supernatant was collected by centrifuged at 12,000 × *g* at 4 °C for 20 min, precleared with Protein G-conjugated Sepharose beads (GE Healthcare) at 4 °C for an hour, and incubated with antibody to Mcl-1 or control IgG at 4 °C for overnight. The complex was precipitated by Protein G-conjugated Sepharose beads at 4 °C for an hour and washed with lysis buffer. Precipitates were separated by boiling in sample buffer at 95 °C for 5 min. Polyubiquitination was detected by western blot analysis with antibody to ubiquitin.

### Statistical analysis

The data are presented as the mean ± SEM obtained from at least three independent experiments. Statistical analysis was performed using unpaired Student’s *t* test or Mann–Whitney *U* test using Prism 8 (GraphPad). *P* values <0.05 was considered as statistically significant.

## Supplementary information

Supplementary Fig S1
