# Supplementary figures and images for: Tubulin acetylation enhances lung cancer resistance to paclitaxel-induced cell death through Mcl-1 stabilization
Source: Cell Death Discov. 2021 Apr 6;7:67. doi: 10.1038/s41420-021-00453-9 (PMC8024319; doi:10.1038/s41420-021-00453-9)

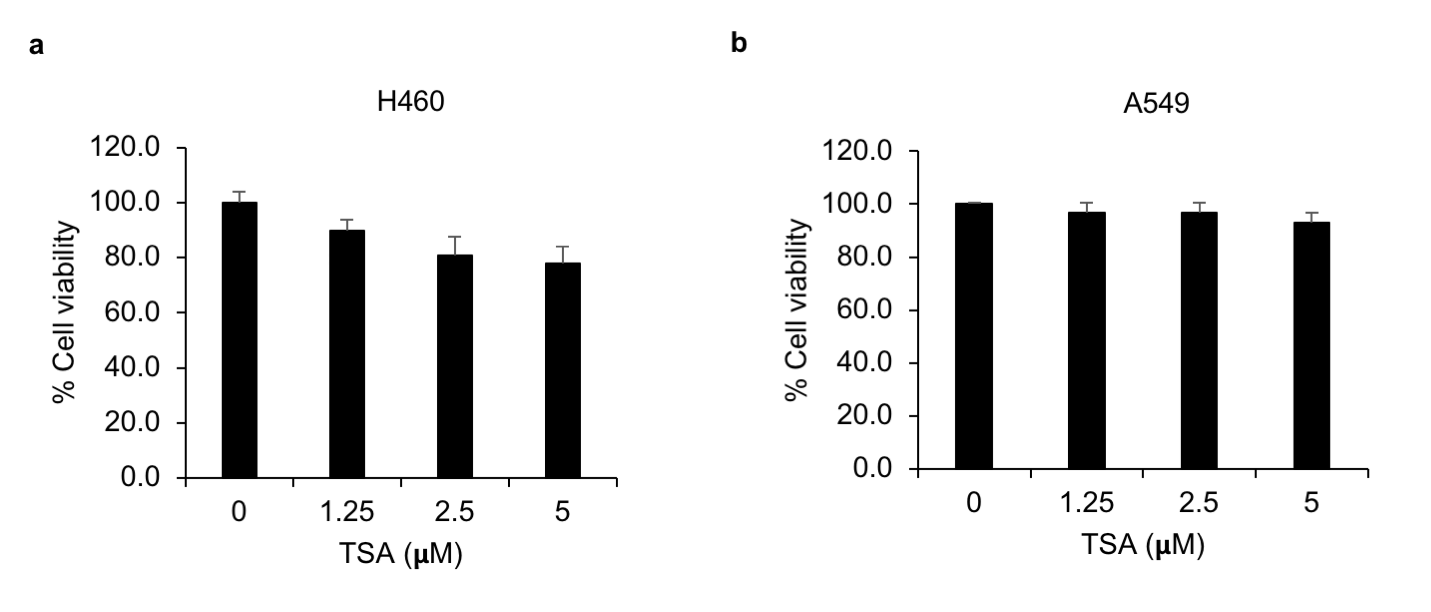

Supplement: Supplementary file 1 — Supplementary Fig S1 [file 41420_2021_453_MOESM1_ESM.tif]
